# Safety of Performing Spirometry During Pregnancy: A Systematic Review

**DOI:** 10.3390/arm94020017

**Published:** 2026-03-06

**Authors:** Zofia Potocka, Katarzyna Górska, Radosław Ciesielski, Dorota Bomba-Opoń, Mirosław Wielgoś, Piotr Korczyński

**Affiliations:** 1 Department of Pulmonary Diseases, Internal Medicine, Thoracic Oncology and Transplantology, National Medical Institute of the Ministry of the Interior and Administration, 02-507 Warsaw, Poland; 2 Department of Obstetrics and Perinatology, National Medical Institute of the Ministry of the Interior and Administration, 02-507 Warsaw, Poland

**Keywords:** asthma, lung function test, pregnancy, safety, spirometry

## Abstract

**Highlights:**

**What are the main findings?**

- There are no studies examining the safety profile of spirometry during pregnancy.
- None of the studies which evaluated spirometry attempts by pregnant women reported any adverse events during the procedure

**What are the implications of the main findings?**

- Further studies are needed to evaluate the safety profile of spirometry during pregnancy.

**Abstract:**

**Introduction**: It is estimated that up to 75% of pregnant women complain of dyspnea at some point during pregnancy. Asthma is the most common chronic pulmonary disease complicating pregnancy. Well controlled asthma does not affect pregnancy negatively. However, asthma exacerbations are linked with several adverse perinatal outcomes. As diligent treatment of asthma significantly reduces the number of asthma exacerbations, it is important to properly detect asthmatic patients among pregnant women in order to provide them with better care. The most efficient way to diagnose asthma is to perform spirometry with a reversibility test. There are no studies that have examined the safety of performing spirometry and, more specifically, a reversibility test, during pregnancy. **Objectives**: In this systematic review we aimed to review current available data regarding the safety of performing spirometry and a reversibility test during pregnancy. **Patients and methods**: For this systematic review, we searched PubMed, Scopus and Cochrane databases. We used the following search terms: (pregnancy); (spirometry); (lung function test); (pulmonary function test); (reversibility test); (post-bronchodilator challenge); (safety). **Results**: We collected reports of spirometry performed on pregnant women and analyzed them for complications that occurred during the procedure. Out of 13,594 records identified for the aforementioned search words, we included 78 documents that met the inclusion criteria. In total, the studies consisted of over 33,405 spirometry attempts performed by 10,617 pregnant women. Additionally, the reversibility test was conducted in nine studies. In all of the selected articles, there were no reports of adverse events occurring while performing spirometry. **Conclusions**: In this systematic review we aimed to summarize the current available data about the safety of performing spirometry during pregnancy. Several studies have investigated pulmonary function tests during pregnancy. No studies reported any adverse events that occurred while performing the procedure. In order to better characterize the safety profile of spirometry, including during pregnancy, further prospective studies systematically reporting on adverse symptoms during spirometry are required.

## 1. Introduction

Rationale: Dyspnea is one of the most common symptoms to be reported among pregnant women. It is estimated that up to 75% of pregnant women will complain of dyspnea at some point during pregnancy [1,2]. In majority, it comes from physiological adaptations the body undergoes in order to sustain a growing fetus. However, when dyspnea is accompanied by a cough or when it persists at rest and during the night further investigation is needed. Pulmonary function tests provide valuable findings to better understand the pathophysiology behind dyspnea. They help to establish a diagnosis of obstructive diseases, including asthma [3]. The prevalence of asthma during pregnancy varies depending on the population. American studies report that from 3 up to 8% of pregnant women have asthma [4]. A Danish study estimated that even up to 12% of pregnant women suffer from asthma [5]. Maternal asthma is linked with several adverse outcomes, both for the mother and the fetus [6,7,8,9]. For example, asthmatic women are more likely to develop preeclampsia, placenta previa and to undergo caesarean section, while for the infant, maternal asthma increases the risk of preterm birth, low birth weight, small-for-gestational age and congenital abnormalities [10,11,12]. As such, it is crucial to recognize pregnant women with asthma to provide better care and minimize the risk of adverse outcomes. In the past, pregnancy was considered a contraindication for lung function tests, and in current standards, late-term pregnancy remains a relative contraindication [13]. We therefore reviewed the published literature on the safety of performing spirometry during pregnancy.

Current guidelines for spirometry are based on a joint statement of American Thoracic Society and European Respiratory Society from 2019. In the report, late-term pregnancy is listed as one of the relative contraindications for spirometry testing. The reasoning behind this statement is linked to an increase in intra-thoracic or intra-abdominal pressure. There is, however, no source material to verify the origins of this annotation. The section on patient details lists age, height, weight and ethnicity as factors to be considered. There is no mention of pregnancy being a critical variable. Norms for the spirometry readings come from the European Respiratory Society Global Lung Function Initiative (GLI) document first published in 2012, later updated in 2022 [14]. In this paper, after reviewing 97,759 records obtained from healthy, non-smoking individuals, of which 55.3% were women, the reference ranges were established. The data were evaluated in relation to age, ethnicity and height. There is no mention of pregnancy being considered, either as a contraindication for the procedure or a variable affecting the spirometry result. Furthermore, a more general document considering pulmonary function tests as a whole was published by the two organizations in 2022 [15]. In this document pregnancy is mentioned only once, in a section addressing special considerations for lung volumes. It states that a pregnancy may affect lung volumes and therefore spirometry results obtained during pregnancy should be interpreted with caution. There is no elaboration on how pregnancy alters lung function. Linked source material leads to two papers. First, a research paper [16] detailing physiological and anatomical changes that a woman undergoes during pregnancy. Secondly, a cross-sectional study published in 2002 by McAuliffe et al. [17] which compares spirometry results obtained from women with singleton and twin pregnancy. There is no mention of the safety of performing spirometry while pregnant.

Objectives: To evaluate the safety of performing pulmonary function tests during pregnancy. We paid special attention to reports including the application of the reversibility test. Additionally, we searched for an answer to the question of whether multiple pregnancy poses a greater risk of adverse outcomes occurring during spirometry than a singleton pregnancy.

## 2. Materials and Methods

For this systematic review we followed the guidelines from the PRISMA 2020 statement (Supplementary Materials). The systematic review has not been registered in any database. Two independent investigators searched PubMed and Cochrane databases, Google Scholar, and forward and backward citations for studies published between the database search and 1 January 1970. We used the following search terms: (pregnancy); (spirometry); (lung function test); (pulmonary function test); (reversibility test); (post-bronchodilator challenge); (safety).

Eligibility criteria: We included documents reporting spirometry results obtained from pregnant individuals. We considered reports published in English, Polish, French, German and Spanish.

Selection process: First, we selected studies based on their titles, with eligibility criteria being on the topic of pulmonary function tests, pregnancy, asthma, or cystic fibrosis. Then, we skimmed through the abstracts of selected works and excluded those that did not involve spirometry being performed by pregnant women. Finally, we read through the full text of the remaining studies. We included studies for which full text was available online. We took note of any adverse events reported as occurring during the procedure. We counted the number of women tested and the number of spirometry tests performed. We paid special attention to studies involving the reversibility test.

All steps, including database search, study selection and study analysis, were performed independently by two investigators.

Study risk of bias assessment: The risk of bias in our study comes from the assumption that not reporting complications that might have happened during a pulmonary function test means that no complications occurred. The second limitation stems from the fact that the majority of studies we analyzed performed the objectives on otherwise healthy subjects, excluding patients with chronic illnesses, smokers or women with complicated pregnancies.

Effect measures: Safety, by the definition from the Merriam-Webster Dictionary, is ‘*1: the condition of being safe from undergoing or causing hurt, injury, or loss*’. In our review by safety we understood not experiencing any potential harms or risk, both to the mother and the fetus, during spirometry. Recognizing that there are a number of possible complications, we preemptively grouped them into categories based on the severity of the potential adverse events. The possible outcomes we suspected to occur were general complications that are expected to happen during procedure in otherwise healthy, non-pregnant subjects. These adverse events include:

- Respiratory alkalosis as a result of hyperventilation;
- Hypoxemia in a patient whose oxygen therapy has been interrupted;
- Chest pain;
- Fatigue;
- Paroxysmal coughing;
- Bronchospasm;
- Dizziness;
- Urinary incontinence;
- Increased intra-cranial pressure;
- Syncopal symptoms [18].

We propose a low-risk category for these complications that routinely occur even in the otherwise healthy population. Furthermore, we identified a high-risk category for adverse events that may require medical treatment or hospitalization.

Synthesis methods: Given the broadness of the subject investigated we categorized each document assessed for eligibility into a category as follows:

1. Prospective longitudinal reports researching pulmonary function findings during the course of pregnancy;
2. Cross-sectional studies examining women in groups of a certain trimester;
3. Single spirometry measurement in a pregnant participant;
4. Case reports.

In the first category we placed 28 studies. We recognized 19 prospective cross-sectional studies. There were 23 studies in which a single measurement of spirometry was performed, without dividing women into trimester groups. In the fourth category we found 8 case reports. We prepared graphs including the number of women participating in the study and the number of spirometry attempts obtained in the study. To meet the inclusion criteria, the study had to state the number of women involved in a study, gestational age they were examined in and the number of spirometry tests performed. It was not possible to conduct a meta-analysis as the studies we reviewed did not report any adverse events.

## 3. Results

**Figure 1:**
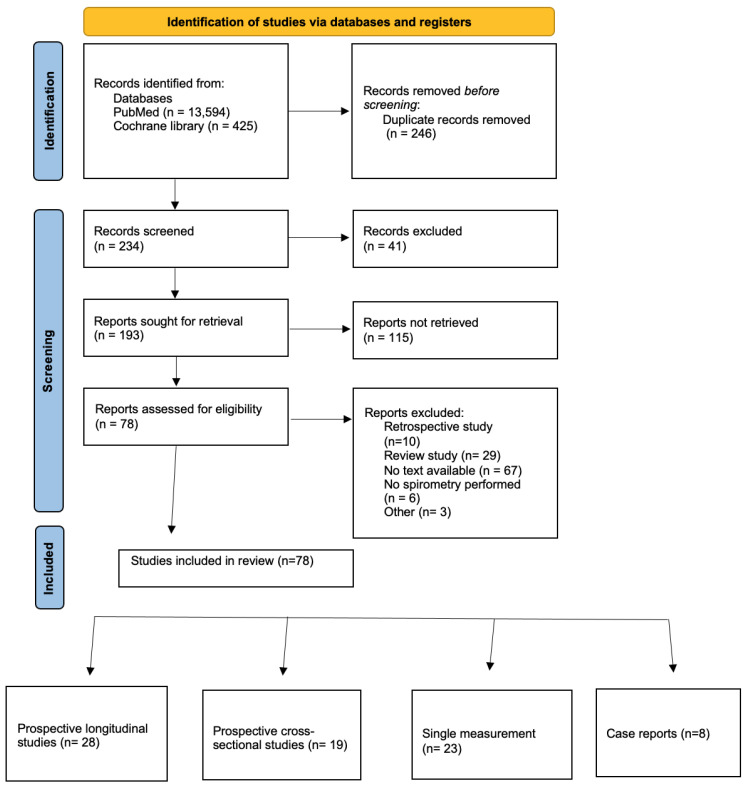
Selection process flow chart.

For this analysis we identified nearly 14,000 results. After initial retrieval we screened 234 records, from which we excluded 41 based on being published before 1970 or being published in a different language than considered. Ultimately, 78 papers met the criteria for final inclusion in this analysis (Figure 1). There were no studies whose main objective was to examine the safety profile of spirometry during pregnancy.

We excluded studies that assessed pregnant women without performing a spirometry, review papers summarizing the topic of pregnancy-related dyspnea and retrospective studies examining historical findings, as there was no possibility for an adverse event to occur. We decided not to include papers that had no full text available. There were three studies that did not fit into any of the categories. One did not report the number of participants [19], the second paper examined remote spirometry [20], which raised concerns about whether the procedure was performed correctly and whether any adverse events would have been reliably reported in the absence of medical supervision. Finally, the third paper did not state the gestational age of the participants during testing and number of spirometry attempts obtained [21]. Further information available in Appendix A.

### 3.1. Prospective Longitudinal Studies

**Figure 2:**
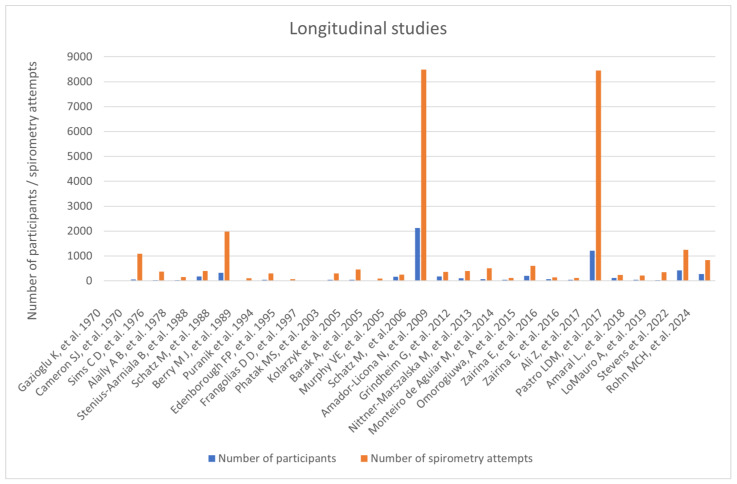
Prospective longitudinal studies [22,23,24,25,26,27,28,29,30,31,32,33,34,35,36,37,38,39,40,41,42,43,44,45,46,47,48,49].

In our search we recognized twenty-eight prospective longitudinal studies that examined women throughout their pregnancy. In total 5973 women participated in those studies, with 27,688 spirometries performed. Thirteen studies examined women with asthma, three studies involved patients with cystic fibrosis and one study assessed women with cardiovascular diseases. Eleven studies were conducted in a healthy population, with consideration of smoking, parity and obesity as additional factors. Across reviewed longitudinal studies there were three papers where the reversibility test was performed. In total, 551 women underwent the examination. None of those studies reported any adverse events occurring during the procedure (Figure 2).

### 3.2. Prospective Cross-Sectional Studies

**Figure 3:**
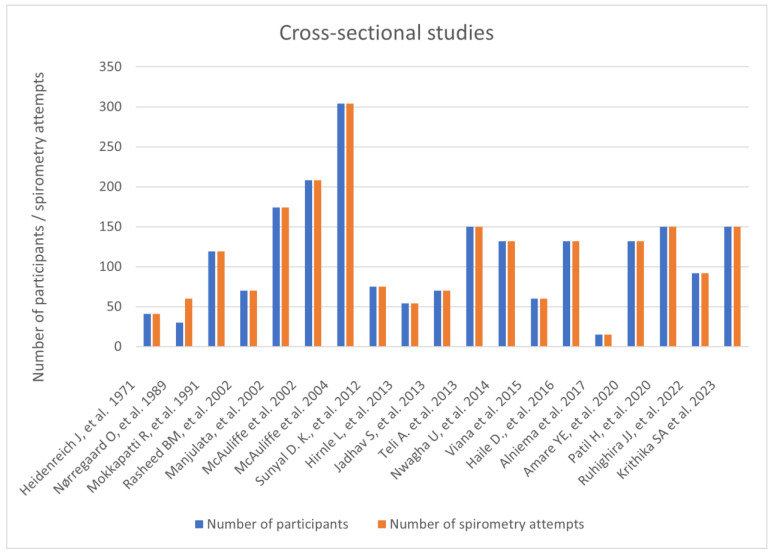
Prospective cross-sectional studies [17,50,51,52,53,54,55,56,57,58,59,60,61,62,63,64,65,66,67].

We found nineteen cross-sectional studies, in which participants were divided into groups based on the trimester they were examined in. Combined, there were 2158 women participating in those studies, with over 2188 procedures performed. There was one publication whose main objective was to evaluate pulmonary function by spirometry in women with high-risk pregnancies. It assessed 30 women with gestational diabetes and/or preeclampsia. The study did not report any adverse events to happen during spirometry in this population. One study from 2002 by McAuliffe et al. [17] compared singleton and twin pregnancies, examining 68 women with multiple pregnancies to 140 women with singleton pregnancies. None of the cross-sectional studies we considered mentioned having performed the reversibility test (Figure 3).

### 3.3. Studies with a Single Measurement of Spirometry

**Figure 4:**
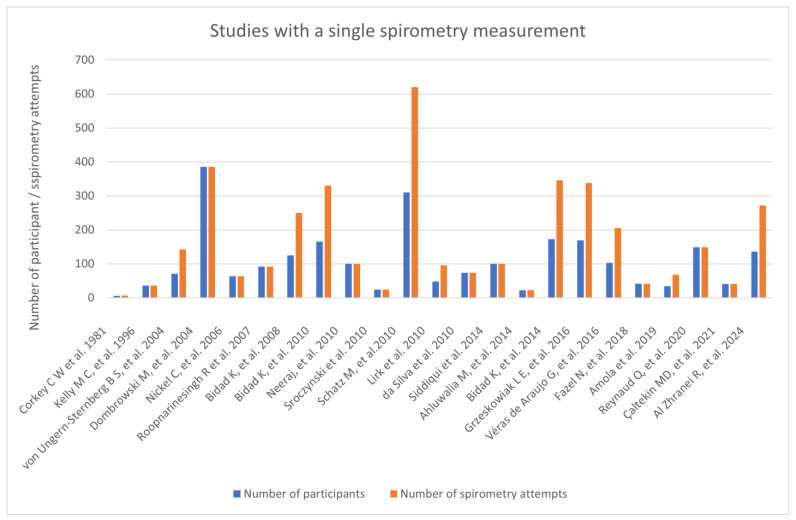
Studies with a single spirometry measurement [22,23,24,25,26,27,28,29,76,77,78,79,80,81,82,83,84,85,86,87,88,89,90].

In our research we gathered twenty-three studies in which a single measurement of spirometry was performed on pregnant participants, without differentiating them by the trimester. Across those studies 2469 women were examined, with 3494 spirometry assessments performed. The difference in number comes from eight papers. First, a study that compared lung function tests in various positions, where each participant was examined sitting and standing [68]. Secondly, from the study that researched the impact of spinal anesthesia on respiratory function during caesarean section, where each participant performed baseline spirometry before induction of the anesthesia and shortly after [69]. We also included in this category a study that compared pulmonary function test results before and after implementing inspiratory muscle training and diaphragmatic breathing exercises for four weeks [70]. Lastly, five papers conducted a reversibility test, which gave two spirometry readings per participant. In nine of the studies women with asthma were examined; three studies involved participants with cystic fibrosis. Aside from the previously mentioned study examining women in late-term pregnancy there were two additional studies that performed lung function tests shortly before delivery by a caesarean section—a study by Lirk et al. [71] and a study by Kelly et al. [72] Another study that examined women in late-term pregnancy was a study by Çaltekin et al. [73] which assessed the relationship between maternal spirometry readings with umbilical cord blood gases values. Despite performing lung function tests in late-term pregnancy, no adverse effects were reported to happen during those procedures. Two studies researched lung function in women with preeclampsia—Roopnarinesingh et al. [74] and da Silva et al. [75] They did not report any adverse events to happen during spirometry. Among twenty-three considered single-measurement studies there were five which included performing the reversibility test on the participants. In these studies, 735 women were tested, and there was no report of complications happening while conducting the procedure (Figure 4).

### 3.4. Case Reports

**Figure 5:**
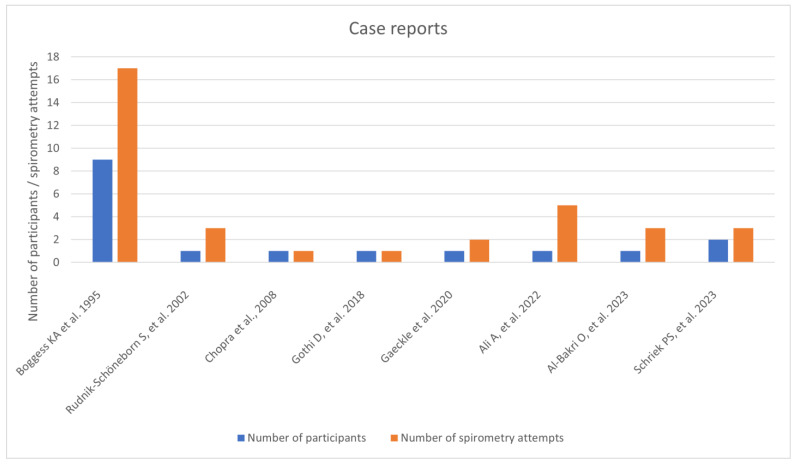
Case reports [91,92,93,94,95,96,97,98].

For our analysis we considered eight case reports that mentioned performing pulmonary function tests on pregnant women. In these papers seventeen women were examined, with combined 38 spirometry assessments obtained. The first study involved a woman with infantile spinal muscular atrophy type II, the second study described a woman with a motor neurone disease. The third described a patient with alpha-1 antitrypsin deficiency. The fourth paper presented successful pregnancies in nine women with interstitial and restrictive lung disease. Two documents presented cases of women with Swyer–James–MacLeod syndrome. The seventh paper involved a patient with a chronic obstructive pulmonary disease, and finally, the eighth paper reported two cases of new onset asthma during pregnancy. In this last document there is a mention of the reversibility test being conducted. None of the papers reported any adverse events occurring during pulmonary function tests (Figure 5).

After an initial search we screened 234 papers regarding lung function tests among the pregnant population. Of these, we selected 78 documents that met the inclusion criteria for this analysis. After reviewing the material, we did not find any information on adverse events during spirometry. There are, however, two considerations that need to be acknowledged. Firstly, not reporting complications does not necessarily indicate that none occurred. It is possible that mild symptoms, which may occur even in healthy individuals, were not described. Secondly, many studies were conducted in otherwise healthy women. Thirty-eight studies were carried out in populations with an additional diagnosis. Twenty-three studies included women with asthma, six examined women with cystic fibrosis, one included patients with gastroesophageal reflux, and one considered women with a cardiopulmonary disease. Among case reports there were women with an infantile spinal muscular atrophy type II, with a motor neurone disease, alpha-1 antitrypsin deficiency, with Swyer–James–MacLeod syndrome, as well as patients with interstitial or restrictive lung diseases and with a chronic obstructive pulmonary disease. None of the papers reported any complication occurring during spirometry in pregnant women.

In the process of this systematic review, we did not find any studies whose main objective was to examine the safety profile of performing spirometry during pregnancy. Our interpretation of the gathered material comes with the assumption that not reporting any adverse events to happen during the procedure means that no serious complications occurred. This, however, creates lack of certainty in concluding that a spirometry is safe to conduct in a pregnant woman, highlighting the need for further research, with a prospective study evaluating any symptoms reported by a pregnant woman during spirometry.

## 4. Discussion

Spirometry is a valuable test offering insight into the possible pathomechanism of dyspnea reported by the patient. It is a safe, non-invasive procedure with a few potential complications. During the examination due to the forced exhalation there is an increase in intra-cranial, intra-thoracic and intra-abdominal pressure. As such, it may pose a risk to the thoracic and abdominal organs. This observation led to late-term pregnancy being a relative contraindication for performing spirometry as listed by the joint statement by the ERS/ATS [13]. In our systematic review we considered the question whether spirometry is safe to perform during pregnancy. We paid special attention to the use of the reversibility test. Additionally, we considered whether multiple pregnancies posed a greater risk of complications happening while performing lung function tests. We collected studies that examined pregnant women with a spirometry and found no reports of adverse events occurring while performing spirometry on a pregnant individual. Among studies included in this review we counted 33,405 spirometry procedures attempted by 10,617 women. These women included both healthy individuals and patients with preexisting conditions. As asthma is the main indicator for spirometry, whether to verify the diagnosis with a bronchodilator testing or to monitor a pre-established disease, studies conducted in women with asthma require special attention. Twenty-three papers considered for this review were conducted in women with asthma, ten of them presented longitudinal studies that monitored pregnant asthmatic women with spirometry during the course of their pregnancy. Nine studies included a bronchodilator challenge by the participants. In one of these papers there is explicit information that the reversibility test was performed 4 h after bronchodilator use; however, in the remaining studies no such information is provided. None of those studies reported any adverse events during the procedure. Nine studies conducted a reversibility test on the participants and none of them reported any adverse events during the procedure. In two studies women with a multiple pregnancy were examined. There was no mention of any complications occurring in that population while performing the spirometry. Among the studies considered for this review, there were 4596 women examined in the first trimester, 6540 women tested in the second trimester, and finally, 7388 women in the third trimester. A total of 5973 women participating in longitudinal studies were examined multiple times during pregnancy. Three studies researched the respiratory functions of women undergoing caesarean section. They included performing spirometry just before the operation, in a highly late-term pregnancy. The gestational age over a certain week was not the exclusion criteria for any of the studies. Conversely, twenty-three studies included women in late-term pregnancy, examining women in the gestational age of over 39 weeks pregnant. Two studies included patients with preeclampsia, one study researched women with high-risk pregnancies. None of these studies reported any adverse events during lung function testing.

We had identified several limitations. First, although the studies did not report any adverse events during the procedure, the safety of a spirometry and a reversibility test was not the primary objective of any of the studies. As such, there is a possibility that the documents did not include any complications that might have happened. Additionally, many of the studies excluded patients with preexisting conditions, obtaining results from otherwise healthy individuals who had a lower risk of adverse events. Similarly, multiple pregnancy was often an exclusion criterion for participation in a study. Therefore, it is not well evaluated whether a multiple pregnancy poses a greater risk of adverse events during spirometry.

The review process had some limitations. We included articles published only in English, Polish, French, German and Spanish; excluding other languages such as Chinese, Bulgarian, Slovak to name a few, of which there have been some papers available. We did not register our study in any databases.

Dyspnea is one of the most common complaints among pregnant women. The majority of these cases stem from the physiological changes that the female body undergoes to sustain a pregnancy. However, there is a significant percentage of women who suffer from asthma. If untreated, asthma can negatively impact the pregnancy, leading to a number of complications. Maternal asthma has been associated with infant complications such as low birth weight, small-for-gestational age, congenital abnormalities and increased infant hospital stay, as well as adverse maternal outcomes including preeclampsia, placenta previa, caesarean delivery and prolonged hospital stay [10,11,12]. Spirometry is a simple, non-invasive and widely available diagnostic test that helps to establish a diagnosis of asthma. Numerous studies have examined pregnant women using spirometry without reporting any complications occurring during the procedure. However, the safety of performing spirometry by a pregnant woman was not the objective of any of the studies. This suggests the need for further research prospectively reporting on adverse events that happen during spirometry.

## Data Availability

In the process of writing this systematic review no new data was created. All analyzed data belong to the authors.

## Supplementary Materials

The following supporting information can be downloaded at: https://www.mdpi.com/article/10.3390/arm94020017/s1, Table S1: PRISMA Checklist [99].

## Abbreviations

The following abbreviations are used in this manuscript:

ATS	American Thoracic Society
ERS	European Respiratory Society
GLI	Global Lung Function Initiative
PRISMA	Preferred Reporting Items for Systematic Reviews and Meta-Analyses

## Appendix A

Information sources: On 18 October 2025 we conducted a search on PubMed database using search words

1. (pregnancy) and (spirometry);
2. (pregnancy) and (lung function test);
3. (pregnancy) and (pulmonary function test);
4. (pregnancy) and (reversibility test);
5. (pregnancy) and (post-bronchodilator challenge);
6. (pregnancy) and (spirometry) and (safety);
7. (pregnancy) and (lung function test) and (safety);
8. (pregnancy) and (pulmonary function test) and (safety);
9. (pregnancy) and (reversibility test) and (safety);
10. (pregnancy) and (post-bronchodilator challenge) and (safety).

On 16 November 2025 we conducted a search in Cochrane Library using the same search words.

Data collection process: The PubMed database was searched using the advanced search tool. The fixed search word was “pregnancy” which was sequentially combined with the terms: “spirometry”, “lung function test”; “pulmonary function test”, “reversibility test”; “post bronchodilator challenge”. Then, a three word search was conducted by adding a second fixed word: “safety”. Next, we performed a similar search on the Cochrane Library database. After gathering the retrieved papers, we continued with the selection process. First, after reviewing each title, we selected publications regarding lung function tests, pregnancy, asthma and cystic fibrosis. Then, we read through the abstracts and chose studies in which spirometry was performed on pregnant women.

Eligible papers included documents performing pulmonary function tests, specifically, spirometry, by pregnant women. The spirometer used in each study should have been validated for the assessment of pulmonary function, but omission of the characteristics of the equipment used in a study was not an exclusion. No restrictions were placed on the reported number of attempts of pulmonary function test attempts.

We collected data on:

- The report: Author, year, and source of publication;
- The study: Objective; methods; study location;
- The participants: Age; race; singleton vs. multiple pregnancy; nulliparous vs. multiparous.

In the papers excluded on the final selection step we recognized additional categories as follows: (V) review papers (VI) no spirometry performed (VII) retrospective studies (VIII) no text available (IX) others. We screened 29 review papers, 6 studies in which no spirometry was performed and 10 retrospective studies. We excluded 68 articles that had no full text available. There were three studies that did not fit into any of the categories. One did not report the number of participants, the second paper examined remote spirometry which raised concerns about whether the procedure was performed correctly and whether any adverse events would have been reliably reported in the absence of medical supervision. Finally, the third paper did not state the gestational age of the participants during testing and number of spirometry attempts obtained.

**Table A1:** List of longitudinal studies included in this systematic review.

Title	Year of Publication	Authors	Number of Participants	Number of Spirometry Tests	Adverse Events During Spirometry
Pulmonary function during pregnancy in normal women and in patients with cardiopulmonary disease [22]	1970	Gazioglu K et al.	24	24	not reported
Ventilatory function in pregnancy [23]	1970	Cameron SJ, Bain HH, Grant IW.	60	1090	not reported
Lung function tests in bronchial asthma during and after pregnancy [24]	1976	Sims C D et al.	39	375	not reported
Pulmonary ventilation in pregnancy [25]	1978	Alaily A B et al.	38	152	not reported
Asthma and pregnancy: a prospective study of 198 pregnancies [26]	1988	Stenius-Aarniala B et al.	181	396	not reported
The course of asthma during pregnancy, post partum, and with successive pregnancies: a prospective analysis [27]	1988	Schatz M et al.	330	1980	not reported
Pulmonary and ventilatory responses to pregnancy, immersion, and exercise [28]	1989	Berry M J, et al.	12	108	not reported
A longitudinal study of pulmonary function tests during pregnancy [29]	1994	Puranik et al.	50	300	not reported
Outcome of pregnancy in women with cystic fibrosis [30]	1995	Edenborough FP et al.	20	66	not reported
Pregnancy and cystic fibrosis: a case-controlled study [31]	1997	Frangolias D D, et al.	7	21	not reported
A longitudinal study of antenatal changes in lung function tests and importance of post partum exercises in their recovery [32]	2003	Phatak MS et al.	50	300	not reported
Lung function and breathing regulation parameters during pregnancy [33]	2005	Kolarzyk et al.	51	459	not reported
Pregnancies and outcome in women with cystic fibrosis [34]	2005	Barak A et al.	8	88	not reported
Asthma self-management skills and the use of asthma education during pregnancy [35]	2005	Murphy VE et al.	172	253	not reported
Spirometry is related to perinatal outcomes in pregnant women with asthma [36]	2006	Schatz M et al.	2123	8492	not reported
Heart sympathetic activity and pulmonary function in obese pregnant women [37]	2009	Amador-Licona N et al.	178	356	not reported
Changes in pulmonary function during pregnancy: a longitudinal cohort study [38]	2012	Grindheim G et al.	100	400	not reported
Fractioned exhaled nitric oxide (FE(NO)) is not a sufficiently reliable test for monitoring asthma in pregnancy [39]	2013	Nittner-Marszalska M et al.	72	504	not reported
Validation of the Asthma Control Test in pregnant asthmatic women [40]	2014	Monteiro de Aguiar M et al.	40	113	not reported
Effect of parity on FVC and FEV1 during pregnancy [41]	2015	Omorogiuwa, A et al.	200	600	not reported
Telehealth to improve asthma control in pregnancy: A randomized controlled trial [42]	2016	Zairina E et al.	72	144	not reported
A prospective cohort study of pulmonary function during pregnancy in women with and without asthma [43]	2016	Zairina E et al.	40	115	not reported
Determinants of low risk of asthma exacerbation during pregnancy [44]	2018	Ali Z et al.	1208	8456	not reported
Longitudinal study of lung function in pregnant women: Influence of parity and smoking [45]	2017	Pastro LDM et al.	120	240	not reported
Use of the Control of Allergic Rhinitis and Asthma Test and pulmonary function tests to assess asthma control in pregnancy [46]	2018	Amaral et al.	42	214	not reported
Adaptation of lung, chest wall, and respiratory muscles during pregnancy: preparing for birth [47]	2019	LoMauro A et al.	39	351	not reported
Maternal body composition and gestational weight gain in relation to asthma control during pregnancy [48]	2022	Stevens et al.	418	1254	not reported
The Association of Periconception Asthma Medication Discontinuation with Adverse Obstetric Outcomes [49]	2024	Rohn MCH et al.	279	837	not reported
Summary	1970–2024		5973	27,688	not reported

**Table A2:** List of cross-sectional studies included in this systematic review.

Title	Year of Publication	Authors	Number of Women Tested	Number of Spirometry Attempts	Spirometry Adverse Events
Ventilation in pregnancy and post partum [50]	1971	Heidenreich J et al.	41	41	not reported
Lung function and postural changes during pregnancy [51]	1989	Nørregaard O et al.	30	60	not reported
Ventilatory functions in pregnancy [52]	1991	Mokkapatti R et al.	119	119	not reported
Incentive spirometry and PEFR in different phases of pregnancy [53]	2002	Rasheed BM et al.	70	70	not reported
Comparative pulmonary function test parameters in non-tribal and tribal women in singleton first, second, and the third trimester of pregnancy [54]	2002	Manjulata et al.	174	174	not reported
Respiratory function in singleton and twin pregnancy [17]	2002	McAuliffe et al.	208	208	not reported
Respiratory function in pregnancy at sea level and at high altitude [55]	2004	McAuliffe et al.	304	304	not reported
Study of Forced Expiratory Volume in First Second (FEV1) and Ratio of Forced Expiratory Volume in First Second and Forced Vital Capacity in Percentage (FEV1/FVC%) in Pregnant Women [56]	2012	Sunyal D. K. et al.	75	75	not reported
Respiratory function in pregnant women [57]	2013	Hirnle L et al.	54	54	Not reported
Comparative study of pulmonary function tests on different trimesters of pregnancy [58]	2013	Jadhav S et al.	70	70	not reported
Physiological Alterations in Pulmonary functions during Pregnancy: Its application in clinical Scenario [59]	2013	Teli A. et al.	150	150	not reported
Forced Expiratory Volume in 6 s (FEV6) and FEV1/FEV6 Values as a Viable Alternative for Forced Vital Capacity (FVC) and FEV1/FVC Values During Pregnancy in South East Nigeria: A Preliminary Study [60]	2014	Nwagha U et al.	132	132	not reported
Analysis of pulmonary function in high-risk pregnancies: a case-control study [61]	2015	Viana MCC et al.	60	60	Not reported
Trends of lung function indices, arterial blood oxygen saturation and pulse rate among the first and third trimester pregnant women in Addis Ababa, Ethiopia [62]	2016	Haile D. et al.	132	132	not reported
The effects of pregnancy on pulmonary function and respiratory muscles power parameters in Sudanese women [63]	2017	Alniema et al.	15	15	not reported
Evaluation of Pulmonary Function Tests Among Pregnant Women of Different Trimesters in Debre Berhan Referral Hospital, Shoa, Ethiopia [64]	2020	Amare YE et al.	132	132	not reported
Analysis of Pulmonary Function and Serum Progesterone Level during Pregnancy: A Cross-sectional Study [65]	2020	Patil H et al.	150	150	not reported
Spirometry profiles among pregnant and non-pregnant African women: A cross-sectional study [66].	2022	Ruhighira JJ et al.	92	92	not reported
Changes in the pulmonary function parameters during pregnancy [67]	2023	Krithika SA et al.	150	150	not reported
Summary	1971–2003		2158	2188	not reported

**Table A3:** List of studies with a single measurement of spirometry included for this systematic review.

Title	Year of Publication	Authors	Number of Women Tested	Number of Spirometry Attempts	Spirometry Adverse Events
Pregnancy in cystic fibrosis: a better prognosis in patients with pancreatic function? [76]	1981	Corkey C W et al.	6	7	not reported
Respiratory effects of spinal anaesthesia for caesarean section [26]	1996	Kelly M C, et al.	36	36	not reported
Impact of spinal anaesthesia and obesity on maternal respiratory function during elective Caesarean section [23]	2004	von Ungern-Sternberg B S, et al.	71	142	not reported
Randomized trial of inhaled beclomethasone dipropionate versus theophylline for moderate asthma during pregnancy [77]	2004	Dombrowski M, et al.	385	385	not reported
Pregnant women with bronchial asthma benefit from progressive muscle relaxation: a randomized, prospective, controlled trial [78]	2006	Nickel C et al.	64	64	not reported
Preeclampsia affects pulmonary function in pregnancy [28]	2007	Roopnarinesingh R et al.	92	92	not reported
Impulse oscillometry in comparison to spirometry in pregnant asthmatic females [79]	2008	Bidad K, et al.	125	250	not reported
Frequency of asthma as the cause of dyspnea in pregnancy [80]	2010	Bidad K et al.	165	330	not reported
Effect of advanced uncomplicated pregnancy on pulmonary function parameters of North Indian subjects [81]	2010	Neeraj et al.	100	100	not reported
Causes of respiratory ailments in pregnancy [82]	2010	Sroczynski et al.	24	24	not reported
The relationship of asthma-specific quality of life during pregnancy to subsequent asthma and perinatal morbidity [83]	2010	Schatz M, et al.	310	620	not reported
Pulmonary effects of bupivacaine, ropivacaine, and levobupivacaine in parturients undergoing spinal anaesthesia for elective caesarean delivery: a randomised controlled study [25]	2010	Lirk et al.	48	96	not reported
Respiratory parameters and exercise functional capacity in preeclampsia [29]	2010	da Silva et al.	74	74	not reported
Pulmonary function in advanced uncomplicated singleton and twin pregnancy [84]	2014	Siddiqui et al.	100	100	not reported
Cystic fibrosis and pregnancy in the modern era: a case control study [85]	2014	Ahluwalia M, et al.	22	22	not reported
Gastroesophagial reflux disease and asthma in pregnant women with dyspnea [86]	2014	Bidad K, et al.	173	346	not reported
An observational study of the impact of an antenatal asthma management service on asthma control during pregnancy [87]	2016	Grzeskowiak L E, et al.	169	338	not reported
Asthma in pregnancy: association between the Asthma Control Test and the Global Initiative for Asthma classification and comparisons with spirometry [88]	2016	Véras de Araujo G, et al.	103	206	not reported
Prospective cohort study of pregnancy complications and birth outcomes in women with asthma [89]	2018	Fazel N, et al.	42	42	not reported
Effect of Inspiratory Muscle Training and Diaphragmatic Breathing Exercises on Dyspnea, Pulmonary Functions, Fatigue and Functional Capacity in Pregnancy during Third Trimester [24]	2019	Amola et al.	34	68	not reported
Pregnancy outcome in women with cystic fibrosis and poor pulmonary function [90]	2020	Reynaud Q et al.	149	149	not reported
The Effect of Maternal Pulmonary Function Test Parameters on Umbilical Cord Blood Gas and the Duration of Labor [27]	2021	Çaltekin MD et al.	41	41	not reported
The impact of body position on vital capacity among pregnant women in the second trimester [22]	2024	Al Zhranei R et al.	136	272	not reported
Summary	1996–2024		2469	3494	not reported

**Table A4:** List of case reports included in this systematic review.

Title	Year of Publication	Authors	Number of Women Tested	Number of Spirometry Attempts	Spirometry Adverse Events
Management and outcome of pregnant women with interstitial and restrictive lung disease [91]	1995	Boggess KA et al.	9	17	not reported
Stable motor and lung function throughout pregnancy in a patient with infantile spinal muscular atrophy type II [92]	2002	Rudnik-Schöneborn S et al.	1	3	not reported
Successful pregnancy outcome in Swyer–James–Macleod syndrome [93]	2008	Chopra et al.,	1	1	not reported
Improvement in spirometry and oxygenation of chronic obstructive pulmonary disease during pregnancy [94]	2018	Gothi D et al.	1	1	not reported
Alpha-1 Antitrypsin Deficiency and Pregnancy [95]	2020	Gaeckle et al.	1	2	not reported
A case of successful pregnancy managed in a patient living with Motor Neurone Disease for more than 3 years [96]	2022	Ali A et al.	1	5	not reported
Swyer–James–MacLeod syndrome in pregnancy: A case report [97]	2023	Al-Bakri O et al.	1	3	not reported
New onset asthma during pregnancy: two case reports [98]	2023	Schriek PS et al.	2	3	not reported
Summary	1995–2023		17	35	not reported

Title

Year of Publication

Authors

Number of Women Tested

Number of Spirometry Attempts

Spirometry Adverse Events
